# Novel translational model of resolving inflammation triggered by UV‐killed *E. coli*

**DOI:** 10.1002/cjp2.43

**Published:** 2016-05-04

**Authors:** Madhur P Motwani, Julia D Flint, Roel PH De Maeyer, James N Fullerton, Andrew M Smith, Daniel JB Marks, Derek W Gilroy

**Affiliations:** ^1^ Centre for Clinical Pharmacology and Therapeutics, Division of Medicine, 5 University StreetUniversity College LondonLondonWC1E 6JFUK; ^2^ Microbial Diseases, Eastman Dental Institute, University College LondonLondonWC1X 8LDUK; ^3^ Centre for Molecular Medicine, Division of Medicine, 5 University StreetUniversity College LondonLondonWC1E 6JFUK

**Keywords:** resolution of inflammation, human *in vivo* model, translational research, neutrophil, macrophage

## Abstract

Whilst numerous studies investigating the aetiology of inflammatory diseases have been performed in rodents, the applicability of these data to human pathophysiology is frequently debated. Regardless of the strengths and weaknesses of rodent models in biomedical research, there is a need to develop models of experimental inflammation in humans. Here, we describe a self‐resolving acute inflammatory response triggered by the intradermal injection of UV‐killed *Escherichia coli* into the forearm of healthy volunteers. Cells and exudates were harvested from onset to resolution by applying negative pressure over the inflamed site. Onset was characterized by high blood flow, neutrophilia and peak levels of pro‐inflammatory cytokines, whilst resolution showed a decline in blood blow, reduction in neutrophils, increase in monocytes/macrophages and waning of classic pro‐inflammatory cytokine levels. An anti‐inflammatory effect, defined as suppression of onset phase events, was demonstrated by administering naproxen, a conventional non‐steroidal anti‐inflammatory drug. In summary, this model of resolving acute inflammation is minimally invasive, highly tractable and allows simultaneous investigation of the vascular response, cellular trafficking and chemical mediator profile of onset and resolution phases of acute inflammation in humans. It can serve as a translational platform to provide mechanistic insights and to test the clinical efficacy of novel anti‐inflammatory and pro‐resolving drugs, and also as a tool in patients to explore inherent defects in resolution pathways.

## Introduction

Mounting a robust inflammatory response to infection or injury is central to host defence and well‐being. However, persistence of this response can lead to a chronic inflammatory state, believed to contribute to the pathology of many diseases in the western world such as cancer, atherosclerosis and rheumatoid arthritis 1. Thus, understanding the factors that control the resolution of the inflammatory response may offer a novel therapeutic approach to treat chronic inflammatory disorders 2. Over the years many rodent models of acute and chronic inflammation have been developed in order to mimic various disease processes 3. Indeed, much of the current anti‐inflammatory pharmacopoeia has been derived from these models, and is largely based on inhibiting factors that drive the acute inflammatory response, such as prostaglandins (non‐steroidal anti‐inflammatory drugs) and TNF‐α (biological therapies) 4.

Whilst animal models of inflammation have generated invaluable information on cell trafficking and cell clearance, as well as the receptors and soluble mediators that control these processes, their relevance to human physiology and pathology has been questioned. This disparity was exemplified by a recent report showing that, although acute inflammatory stimuli such as trauma, burns and endotoxin result in highly stereotyped genomic responses in humans, equivalent responses in mouse models were not comparable 5. Whilst controversial 6, 7, this has led to a call for ‘higher priority for translational medical research to focus on the more complex human conditions rather than relying on mouse models to study human inflammatory diseases 5. However, studying fundamental resolution biology in patients is challenging due to disease heterogeneity as well as co‐morbidities and polypharmacy.

To understand the natural course of resolution of acute inflammation in humans, there is need for a model that not only resembles the inflammatory response seen clinically in disease settings, but also utilizes a uniform dose of inflammatory stimulus to avoid heterogeneity. The model should also facilitate minimally invasive sampling of inflamed tissues and allow the appreciation of the classical signs and symptoms of inflammation.

Acknowledging the above characteristics, we have developed a model that combines and adapts two techniques previously used separately to cause acute inflammation and harvest the inflammatory exudate. The first step involves intradermal injection of *Escherichia coli* killed by ultraviolet light (UV killed *E. coli*/UVKEc) into the dermis of the forearm of healthy volunteers to trigger an inflammatory response in an easily accessible site 8. The second step involves raising a negative pressure suction blister over this site, to obtain the inflammatory exudate from the tissue 9. Here, we present the characterization of this model encompassing the vascular and immunological events during onset and resolution of acute inflammation and discuss the merits of this approach compared to existing models of inflammation. Finally, we highlight the potential translational use of this approach to further understand resolution physiology and pharmacology.

## Materials and methods

### Ethics statement

The study was approved by UCL Institutional Ethics Committee (Project ID: 5051/001). Written informed consent was taken from all the volunteers. All procedures were in accordance with the Helsinki Declaration of 1975, as revised in 1983.

### Volunteers

#### Characterisation study

Twenty four young (18–50 years) healthy, non‐smoking male volunteers were recruited. Volunteer exclusion criteria included any history of chronic inflammatory disease, allergies, recent illness (<1 month), vaccination within the last 3 months, regular medication, or any medication in the preceding week. During the study period, volunteers were asked to refrain from alcohol and heavy exercise.

#### Naproxen study

Three young healthy non‐smoking male volunteers were recruited for this study arm. Enteric‐coated naproxen 500 mg was given twice daily for 3 days prior to UVKEc injection. In addition to the exclusion criteria mentioned above, volunteers with history of hypersensitivity to non‐steroidal anti‐inflammatory drugs (NSAIDs) or gastrointestinal ulceration were excluded from this study arm.

### Ultraviolet light killed *E. coli* (UVKEc): preparation and injection

UV killed *E. coli* (Strain: NCTC 10418, Source: Public Health England, UK) were prepared as described previously 8. Briefly, *E. coli* were grown overnight in Luria Broth (Sigma) at 37°C. The next morning the bacteria were washed twice in sterile PBS (2500 *g*, 20 min, 4°C) and resuspended in a sterile petri dish. Bacteria were then killed by exposure to an ultraviolet light (UV) source (302 nm, ChemiDoc, trans‐UV mode; Bio‐Rad laboratories) for 60 min and then washed again in sterile saline. Bacterial counts were determined by optical density (OD_600_ = 0.365 equates to 10^8^ *E. coli*/ml) 10. UVKEc were resuspended in a volume of sterile saline to obtain the count of 1.5 × 10^8^/ml, aliquoted into sterile eppendorf tubes and then frozen at −80°C until used for injections. Non‐viability of final sample was confirmed by UCLH (University College London Hospitals, UK) Microbiology department.

### Intradermal injection of UVKEc

**Figure 1:**
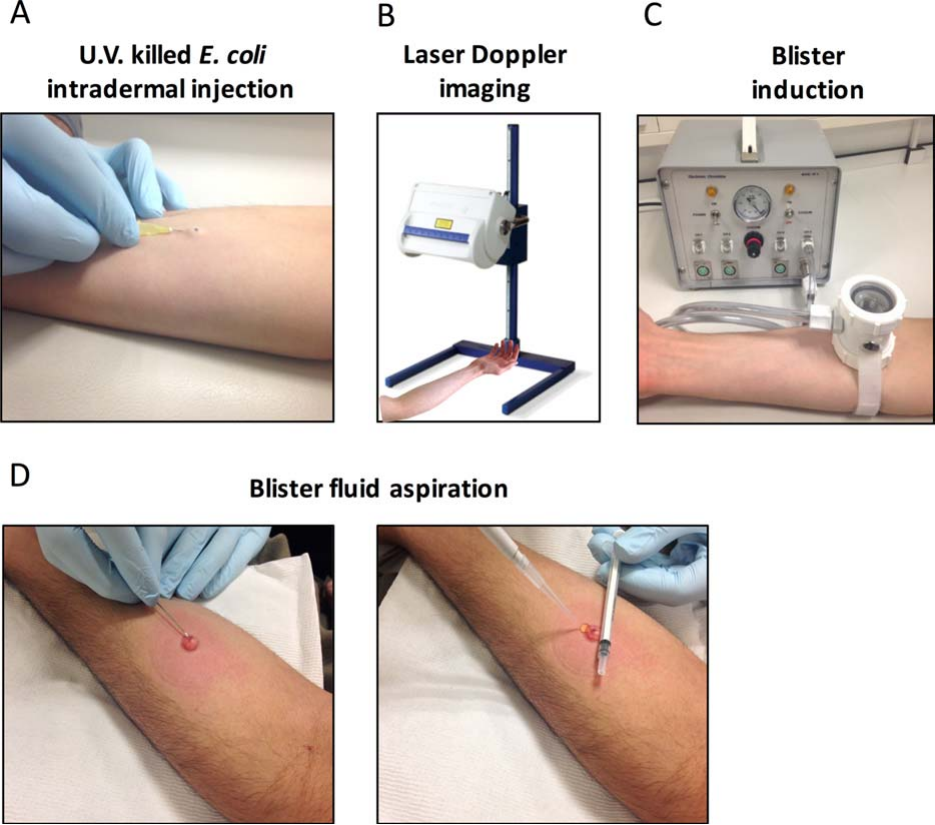
**Study protocol**. Acute inflammation was triggered in the ventral aspect of the forearm of healthy volunteers by the intradermal injection of 1.5 x 10^7^ UV‐killed *E. coli* (UVKEc) suspended in 100 μl of sterile saline (A). At pre‐defined time‐points after injection, the inflamed site was scanned by a laser Doppler imager to assess blood flow (B). Subsequently, a suction chamber connected to a negative pressure instrument was placed over the injection site to raise a 10 mm blister to acquire the inflammatory exudate present at the site (C). When the blister was completely formed, its roof was pierced using a sterile needle and the contents were aspirated (D).

After disinfecting and shaving the skin, 1.5 × 10^7^ UVKEc in 100 μl saline were injected intradermally into a marked site on the volar aspect of each forearm. To characterize the different phases of acute inflammation, each forearm was allotted to one of the predefined time‐points namely 4, 8, 14, 24, 48 or 72 hours (h). Thus inflammation was allowed to progress for the duration of the time‐point after which a suction blister was raised over the marked injection site, and then aspirated immediately. In summary, volunteer had two injection sites, one on each forearm, and contributed to two time points. On a separate group of volunteers, blister was raised on the naïve skin and treated as the baseline time point. Study time‐points were discussed with volunteers before consenting (Figure 1A).

### Laser Doppler imaging

Laser Doppler Imager (moor LDI‐HIR, Moor Instruments Ltd, Axminster, Devon, UK) was used to quantify the blood flow at the site of inflammation. At predefined time points after injection of UVKEc, the forearm was placed under the scanner at a fixed distance to scan a fixed area. The scanner emits a laser beam, a portion of which is scattered by red blood cells present at the inflamed area. The scattering causes a change in frequency of the reflected light which is then detected by a photo detector. The velocity and concentration of red blood cells at the site directly affect the Doppler frequency shifts and account for the signal strength measured in arbitrary perfusion units. The data was analysed by moorLDI software (Version 5) and displayed as colour coded images showing different blood flow levels over the scanned area. The total blood flow (measured in perfusion units) was calculated as product of number of valid pixels above background signal (Cut‐off = 300 perfusion units) and the mean blood flow signal over the valid pixels 11, 12 (Figure 1B).

### Induction of suction blister

To obtain the inflammatory exudate from the site of inflammation, a 10 mm diameter suction blister was induced directly over the site of injection. This procedure is a modified version of the procedure described previously 9. Briefly, a suction blister was raised by placing a suction blister chamber connected by tubing to a negative pressure instrument (NP‐4, Electronic diversities Ltd., MD, USA). The chamber was made of three parts: an aluminium plate with 10 mm aperture, a nylon cup, and a transparent glass lid, all secured by a detachable air tight seal. The suction chamber was placed on the forearm with the 10 mm aperture centred over the marked injection site. After securely strapping the suction chamber on to the forearm, the negative pressure was applied gradually from 2 to 6–7 inches of Mercury (Hg) until a single uniloculated blister covering the surface area within the aperture was formed. The pressure was brought down gradually to baseline after the blister was completely formed. The suction blister induction process took 1.5–2 h (Figure 1C).

### Blister exudate aspiration

The suction blister was aspirated immediately after formation to collect the inflammatory exudate. To aspirate the exudate, the blister roof was pierced along its lateral border using a 26.5 gauge needle. The exudate was then gently pushed out onto the skin by rolling a 1 ml syringe over the blister roof and was simultaneously aspirated using a 200 μl pipette tip. The exudate was collected into a well of a 96 well V‐bottom plate containing 50 μl of 3% sodium citrate (Sigma) in PBS (Gibco). The plate was then centrifuged at 1000 *g* for 5 min at 4°C to separate the cells from the supernatant. After centrifugation, the resulting cell pellet was resuspended in 200 μl of ACK lysis buffer (Lonza) to lyse the red blood cells (RBC). The RBC depleted cell pellet was resuspended in 100 μl of cell staining buffer (PBS with 5% FCS (Gibco) + 0.1% sodium azide] and the cell count was obtained using a manual haemocytometer. The supernatant was weighed to estimate the blister fluid volume, split into 30 μl aliquots and then stored at −80°C. The blister area was then cleaned using 0.5% Cetrimide spray (Savlon) and covered with a protective dressing pad (9 × 10 cm, Mepore) 13.

### Peripheral blood analysis

Peripheral blood was collected by venepuncture from the medial cubital vein using an aseptic technique. Blood was collected at baseline, 4, 24, 48 and 72 h after UVKEc intradermal injection into EDTA and heparin anti‐coagulated vacutainers (BD). For full blood counts, EDTA anti‐coagulated blood was sent to an external pathology lab (The Doctor's Laboratory, Whitfield Street, London, UK). Heparin anti‐coagulated blood was centrifuged at 2500 *g*, 10 min, room temperature to separate plasma. Plasma was aliquoted and stored at −80°C until analysed for cytokines.

### Flow cytometry

Leucocyte subpopulations in the blister fluid were identified by poly‐chromatic flow cytometry. For cell surface marker staining, blister cells in 100 μl of cell staining buffer (PBS with 5% FCS + 0.1% sodium azide) were incubated with an antibody cocktail described in supplementary material, Table S1. Stained samples were washed in cell wash buffer (PBS with 1% FCS + 2 mm EDTA) at 1000 *g* for 5 min, 4°C. Cells were then fixed in an equal volume of 1% paraformaldehyde and stored in the dark at 4°C and analysed within 4 h on BD LSR Fortessa™ flow cytometer. Flow cytometry data was analysed by Flowjo software (Treestar Inc.)

### Multiplex ELISA

The human cytokine 30‐plex kit was purchased from Meso Scale Delivery (MSD, MD, USA). Each kit consists of three 10‐plex panels – Proinflammatory Panel 1, Cytokine Panel 1 and Chemokine Panel 1. The supernatant from blister exudate or the plasma was diluted in appropriate assay diluent and the assay was performed as per manufacturer's instructions. All assay components were supplied by the manufacturer.

### Statistical analysis

All data are expressed on a logarithmic scale as individual values with medians, apart from cell proportions which are expressed as median percentages. Data from the naproxen study arm are expressed as median with interquartile range and analysed by the Mann‐Whitney U‐test. A *p* value <0.05 was taken as the threshold for significance. Prism 6 (GraphPad Software) was used for statistical analysis.

## Results

### Intradermal injection of UV killed *E. coli* (UVKEc) triggers a clinically discernible and self‐resolving acute inflammatory response

**Figure 2:**
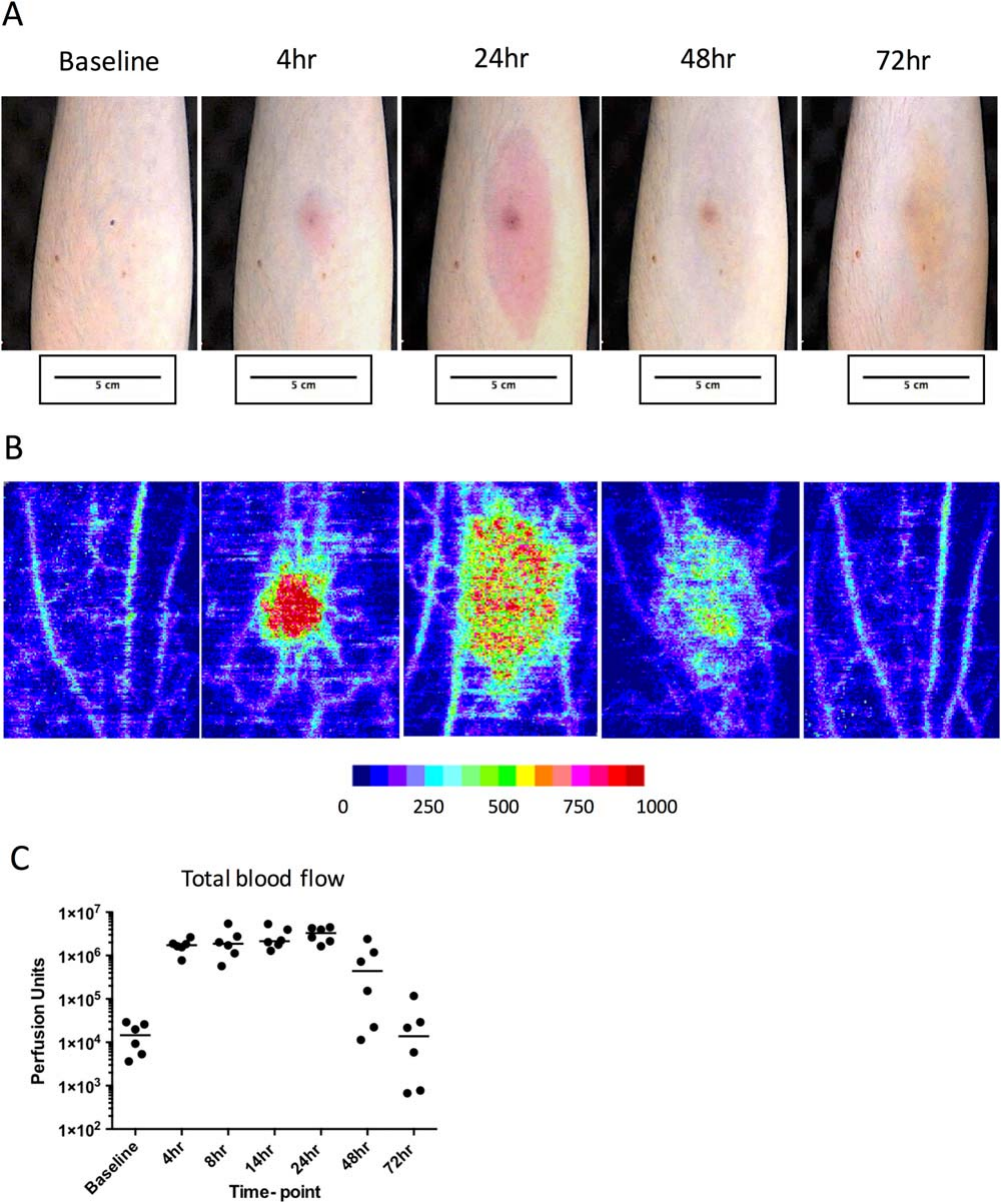
**Assessment of vascular response at the site of UVKEc‐triggered resolving acute inflammation**. Acute inflammation was triggered in the ventral aspect of forearm of healthy volunteers by the intradermal injection of 1.5 x 10^7^ UV‐killed *E. coli* (UVKEc) suspended in 100 μl of sterile saline. Vascular response at the site was assessed by laser Doppler imager (moorLDI‐HIR) which captured the camera images (A) and also generated the flux images (B). Representative flux images at baseline, 4, 24, 48 and 72 h are shown here. Flux images were analysed by moorLDI software to quantify total blood flow. Total blood flow, measured in perfusion units, is shown in panel C. Data are expressed as individual values with median; *n* = 6 at each time point.

Within 4 h of UVKEc injection, the injection site became red; this increased in size until 24 h and then faded by 72 h (Figure 2A). To quantify this vascular response objectively, the site was scanned by a laser Doppler imager. Figure 2B shows the representative laser Doppler images. Figure 2C shows the total blood blow in perfusion units at each time point, indicating that blood flow plateaued from 4 h until 24 h, followed by a decline at 48 h before returning to baseline levels by 72 h.

### Cellular composition of the inflammatory exudate at the site of UVKEc‐triggered resolving acute inflammation

**Figure 3:**
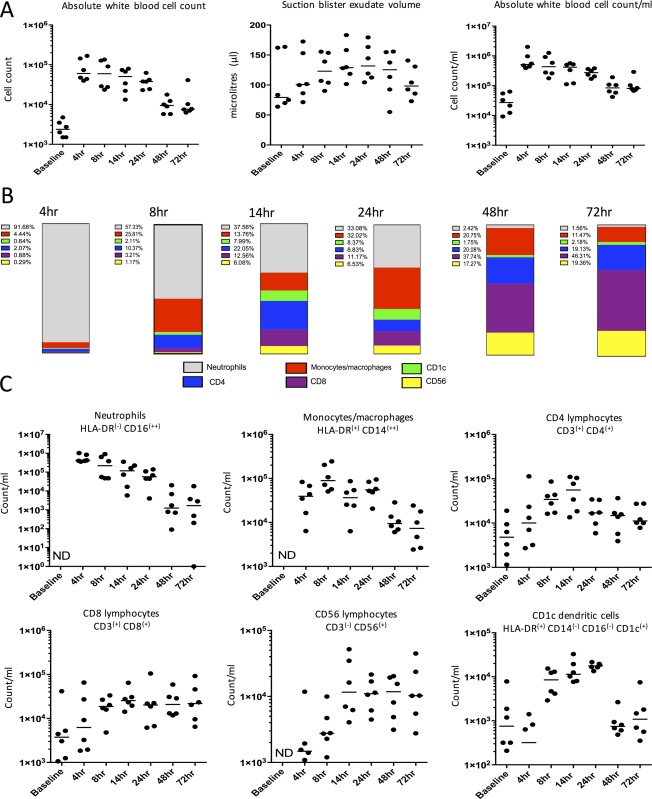
**Cellular profile at site of UVKEc‐triggered resolving acute inflammation**. A suction blister was raised over the inflamed site by negative pressure to collect the inflammatory exudate. Exudate was centrifuged to separate cells from the supernatant containing soluble mediators. Panel A shows total white blood cells, counted manually by haemocytometer, suction blister exudate volume and total cell count per ml of exudate acquired. Panel B shows the relative proportion of neutrophils, monocytes/macrophages, CD4^+^ and CD8^+^ T lymphocytes, CD56^+^ NK cells and CD1c^+^ dendritic cells calculated at each time point, using the gating strategy illustrated in Figure 4. Panel C shows cell count/ml of the above cell populations at different time points. Data are expressed as individual values with median; *n* = 6 at each time point. ND = not detectable.

To interrogate the cellular and soluble mediator profile during different phases of the inflammatory response, inflammatory exudate was obtained from the site by raising a suction blister over it. Inflammatory exudate was centrifuged to separate cells from soluble mediators. Figure 3A displays the temporal profile of total white blood cells, which peaked at 4 h and declined progressively up to 72 h post injection. It also shows the volume of inflammatory exudate acquired at each time point and the cell count adjusted for exudate volume, expressed as cells/ml.

**Figure 4:**
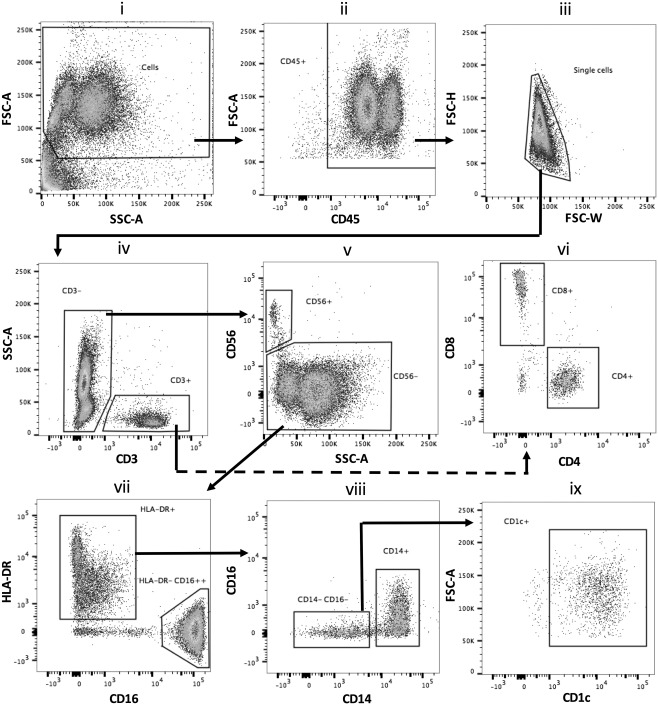
**Polychromatic flow cytometric gating strategy to identify subtypes of immune cells at the site of UVKEc‐triggered resolving acute inflammation**. Inflammatory exudate obtained using negative pressure suction chamber was centrifuged to pellet cells, which were then incubated with a fluorochrome‐tagged antibody cocktail (supplementary material, Table S1). Stained cells were acquired on BD LSR Fortessa™ and analysed by Flowjo software. The gating strategy employed to identify neutrophils, monocytes/macrophages, CD4^+^ and CD8^+^ T lymphocytes, CD56^+^ NK cells and CD1c^+^ dendritic cells is illustrated. Representative plots from the 14 h time point (when all subtypes of immune cells are adequately present) are shown.

Subpopulations of blister leucocytes were characterized by polychromatic flow cytometry using a gating strategy described previously 14. Figure 4 shows the typical gating strategy employed here to discern the cell types at different time points (a representative sample from the 14 h time point when all immune cell subtypes are adequately present is shown). After exclusion of debris (Figure 4i), cells of haematopoetic origin (CD45^+^) were separated (Figure 4ii) followed by doublet exclusion (Figure 4iii). CD45+ single cell events were probed for CD3 expression and were further separated into CD4^+^ and CD8^+^ T cells (Figure 4iv, vi). The remaining CD45^+^/CD3^‐^ compartment (Figure 4iv; B cells were present in very low numbers in the blister at all the time‐points) was probed for CD56^+^ NK cells (Figure 4v). The CD56^‐^ myeloid cell population was then investigated for HLA‐DR and CD16 to separate mononuclear cells and granulocytes (Figure 4vii). The HLA‐DR^+^ population was further probed for CD14 and CD16, revealing two distinct populations of HLA‐DR^+^/CD14^+^ monocytes/macrophages and HLA‐DR^+^/CD14^‐^/CD16^‐^ cells (Figure 4viii); a major proportion of which comprised CD1c^+^ dendritic cells (Figure 4ix). The HLA‐DR^‐^ gate comprised mainly CD16^++^ neutrophils (Figure 4vii). Figure 3B shows the relative proportions of cells characterized above and Figure 3C shows the count/ml of these cells in blister exudate at different phases of inflammation.

On studying the temporal profiles of the individual cell types (expressed as count/ml of inflammatory exudate), it was evident that the influx of neutrophils at the site peaked at 4 h, which then gradually cleared, reducing by >90% at 48 h. A closer look at monocyte/macrophage kinetics suggested a biphasic profile; a first peak was observed at 8 h and then a rise at 24 h (Figure 3C). Further analysis of cell surface markers on these cells revealed an increase in the expression of CD16 as well as CD163 and CCR7 from 24 to 48 h (supplementary material, Figure S1). In addition to the distinct population of HLA‐DR^+^/CD1c^+^ cells peaking at 24 h, CD1c expression was found to increase on these monocytes/macrophages from 48 h onwards (supplementary material, Figure S1).

Analysis of lymphocyte populations showed the presence of CD4^+^ and CD8^+^ T cells in the naïve skin. The number of these cells increased with the onset of inflammation, but unlike the myeloid cells (neutrophils, monocytes/macrophages and dendritic cells) the number of these lymphoid cells (including CD56^+^ NK cells) remained constant after the resolution of the clinical signs of inflammation. At 48 and 72 h, lymphocytes were therefore the major cell type present in the inflammatory exudate.

To ensure that the systemic inflammatory response to UVKEc injection was minimal and self‐resolving, full blood counts (FBCs) were obtained from peripheral blood. At 4 h, a mild increase in total white blood cells and neutrophils above the reference range (marked as dotted lines on the graph) was observed. By 24 h, total white blood cell and neutrophil counts returned to baseline levels. Monocyte and lymphocyte counts remained within the reference ranges (supplementary material, Figure S4).

### Cytokine and chemokine profile at the site of UVKEc‐triggered resolving acute inflammation

**Figure 5:**
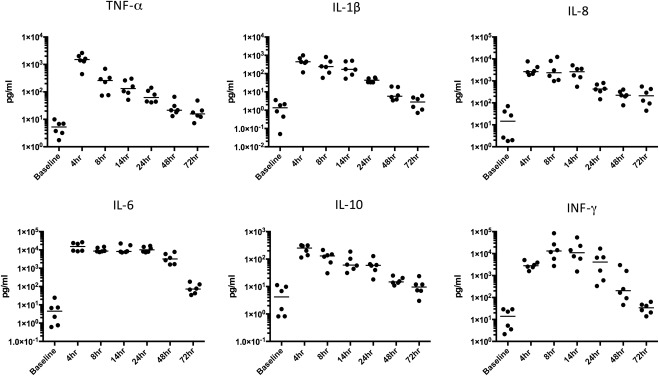
**Cytokine and chemokine profile at the site of UVKEc‐triggered resolving acute inflammation**. Inflammatory exudate obtained using negative pressure suction chamber was centrifuged to separate cells from the supernatant containing soluble mediators. Cytokines and chemokines in the supernatant were probed by a multiplex ELISA. Data are expressed as individual values with median; *n* = 6 at each time point.

The cell‐free inflammatory exudate was analysed for chemokines and cytokines and studied alongside the vascular and cellular response described above. Figure 5 shows that, at 4 h, the classical pro‐inflammatory cytokines TNF‐α, IL‐8, IL‐1β were at their highest concentrations alongside the anti‐inflammatory cytokine IL‐10. IFN‐γ peaked at 8 h, whilst IL‐6 plateaued from 4 h until 24 h. By 72 h, concurrent with the vascular and cellular resolution, the levels of these cytokines had declined to baseline levels. Additional cytokines measured during the multiplex ELISA are shown in supplementary material, Figures S2 and S3. Cytokines were also measured in the plasma but their levels were either very low or did not exhibit the temporal profile observed in blister exudates. (supplementary material, Figure S5).

### Validation of the UVKEc‐triggered resolving inflammation model for testing an anti‐inflammatory effect

**Figure 6:**
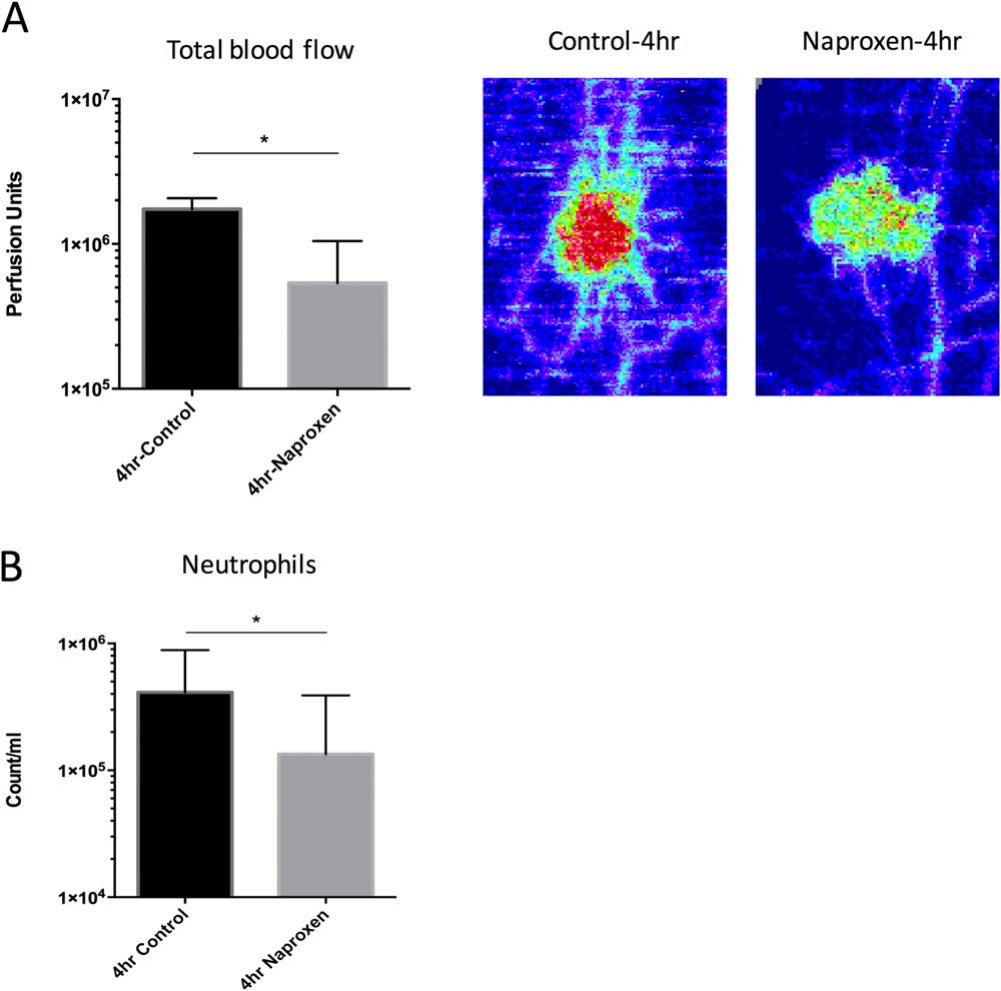
**Validation of the anti‐inflammatory effect of naproxen using the UVKEc‐triggered resolving acute inflammation model**. Volunteers were given naproxen 500 mg twice daily for 3 days prior to the UVKEc injection. Four hours after the injection, the site was scanned by laser Doppler imager to quantify total blood flow and then a suction blister was raised to collect the inflammatory exudate. Panel A shows the total blood flow at 4 h in the control and naproxen study arms. Representative laser Doppler flux images are also shown. Panel B shows the neutrophil count/ml at 4 h between control and naproxen study arm. Data are expressed as median with interquartile range; *n* = 6 for control and *n* = 3 for naproxen study arm, *p < 0.05.

In a separate study arm, three volunteers were given naproxen 500 mg twice daily for the 3 days prior to UVKEc injection, after which a blister was raised at 4 h. At 4 h, naproxen significantly reduced the total blood flow over the injection site as well as the neutrophil count in the blister exudate (Figure 6).

## Discussion

The intradermal injection of UVKEc elicits a self‐resolving inflammatory response that allows for the simultaneous assessment of vascular response, cellular migration and soluble mediator profiles during onset and resolution phases of acute inflammation in humans.

The onset phase was evident within 4 h and was characterized by an increase in blood blow, maximal number of immune cells comprising mainly neutrophils, and the highest concentrations of classical pro‐inflammatory cytokines. The vascular response plateaued from 4 h until 24 h and started to resolve thereafter, approaching baseline levels by 72 h. In contrast, cellular resolution, classically defined by reduction of neutrophil numbers 15, ensued gradually from 4 h, with >90% of neutrophils cleared by 48 h. Unlike neutrophils, monocytes/macrophages showed a biphasic profile. At 8 h, monocytes/macrophages resembled classical phenotype (CD14^hi^ CD16^‐^), followed at 24 h by the presence of a population resembling the intermediate phenotype (CD14^hi^CD16^+^) expressing resolution phase related markers: CD163 16, 17 and CCR7 18, 19. In line with cellular resolution, the pro‐inflammatory cytokines declined gradually, reaching near baseline levels by 48 h. At 72 h, blood flow reverted back to normal, however the inflammatory exudate still contained cells of the adaptive immune system, namely CD4, CD8 and CD56 lymphocytes. These findings are consistent with our existing knowledge of the acute inflammatory response seen in humans and are also reminiscent of events characterizing cellular resolution in mouse models 20, 21. In addition, the expected suppression of the onset phase, at a clinical and cellular level, by the anti‐inflammatory drug naproxen supports the use of this model for pharmacological drug testing.

When compared to other *in vivo* models of acute inflammation in humans, this model offers distinct advantages. Firstly, it generates a robust, clinically evident inflammatory response followed by resolution. This is attributable to our choice of inflammatory stimulus, and its site of inoculation in the dermal connective tissue. Other skin models of inflammation triggered by tissue injury either using a chemical, eg Cantharidin 22 or mechanical trauma eg dermal abrasion 23 or tape stripping 24 generate an inflammatory response evidenced by cell migration and detection of pro‐inflammatory mediators, but are suitable only to study the onset phase. Studying resolution in such tissue injury models has been inadequate as inflammation develops on debrided skin and not within the connective tissue. The connective tissue environment provides the stromal scaffold including the vascular and lymphatic network. This facilitates cell‐cell interaction and cell migration important for key processes leading to resolution ie efferocytosis 25, 26, vascular reverse transmigration 27 and lymphatic emigration 19. Similar to skin tissue injury models, the skin window technique, which involves creating an artificial cavity over the denuded skin site, only serves well to study initial cell migration due to the absence of stromal scaffold 28, 29.

This model of resolving inflammation also utilizes a minimally invasive technique of suction blister to acquire the inflammatory exudate from the site. Obtaining inflammatory exudate in this way provides cells that facilitate polychromatic flow cytometry, which allows simultaneous evaluation of multiple immune cell markers. Furthermore, soluble mediators can be measured in the cell‐free exudate. This contrasts with other models of skin inflammation caused by intradermal endotoxin injection 30, or UV radiation 31, 32 which utilize the relatively invasive technique of skin biopsy in order to study the inflammatory response. The skin biopsy sample thus obtained has been shown to provide limited information either on individual cell populations by semi‐quantitative immunohistochemistry or on individual cytokine measurements by Western blot.

Another commonly used model of acute inflammation involves lung lipopolysaccharide (LPS) challenge 33. Compared to this LPS model, our UVKEc‐triggered model of resolving inflammation is significantly less invasive and also allows interrogation of more than one skin site thus permitting evaluation of different phases of inflammation in the same person. As to the i.v. endotoxaemia model 34, which primarily elicits the systemic inflammatory response mimicking early sepsis, intradermal UVKEc investigates tissue inflammation driven by the circulating and the resident immune cell compartment.

Although we argue that our model of self‐resolving inflammation is well‐suited to the study of resolution of inflammation in humans, it does have some limitations that are important to consider when planning research studies. The robust inflammatory response produced during onset causes a moderately swollen and tender lesion over the forearm. In some cases this is accompanied by minor discomfort in the axillary region, presumably due to increased lymphatic flow in the axillary lymph nodes. Some volunteers also complained of headache and muscle ache in the first 4‐8 h, resembling a mild flu‐like syndrome. However, in all cases the symptoms subsided after 24 h and disappeared by 48 h. The blister induction process in itself caused only mild discomfort. After blister aspiration, the site formed a scab which left a pigmented scar. This faded considerably within 4–6 weeks on Caucasian skin but took longer on darker skin types.

In conclusion, intradermal injection of UVKEc generates a rapid and robust inflammatory response that allows investigation of immunological and vascular events associated with onset and resolution phases of inflammation in a minimally invasive way. We propose that it is a robust translational platform to study the mechanisms of novel anti‐inflammatory and pro‐resolving drugs and simultaneously assess their effect on the clinical signs of inflammation. In addition, it can be used as a tool in patients with chronic inflammatory disorders to investigate the contribution of defective resolution to the pathogenesis of such conditions.

## Author contributions

MPM and DWG designed the study. MPM performed majority of experiments, analysed the data and prepared the figures. JDF helped with data analysis and multiplex ELISA. RPHDM and JNF helped with flow cytometry and suction blister experiments. AMS assisted with bacteria culture and laser Doppler imaging. DJBM helped with multiplex ELISA and naproxen study. DWG and MPM wrote the manuscript.

## Supporting information

SUPPLEMENTARY MATERIAL ONLINE

**Table S1.** Antibody panel for polychromatic flow cytometry

**Figure S1.** Assessment of monocytes/macrophages for resolution phase related cell surface markers

**Figure S2.** Additional cytokines and chemokines measured at the site of UVKEc‐triggered resolving acute inflammation ‐ I

**Figure S3.** Additional cytokines and chemokines measured at the site of UVKEc‐triggered resolving acute inflammation ‐ II

**Figure S4.** Peripheral blood counts in UVKEc‐triggered resolving acute inflammation model

**Figure S5.** Cytokine and chemokine profile of plasma in UVKEc‐triggered self‐resolving acute inflammation model
