# Supplementary material for: Novel translational model of resolving inflammation triggered by UV‐killed E. coli
Source: J Pathol Clin Res. 2016 May 4;2(3):154–65. doi: 10.1002/cjp2.43 (PMC4958736; doi:10.1002/cjp2.43)
Supplement: Supplementary file 1 — Table S1. Antibody panel for polychromatic flow cytometry [file CJP2-2-154-s006.docx]

Supplementary Table 1

| **Table 1 : Antibody panel for polychromatic flow cytometry** | | | |
| --- | --- | --- | --- |
| Fluorescent  conjugate | Cell surface marker | Antibody  clone | Manufacturer |
| FITC | CD3 | HIT3a | Biolegend |
| BV785 | CD45 | HI30 | Biolegend |
| AF400 | CD4 | RPA-T4 | Biolegend |
| BV 510 | CD8 | RPA-T8 | Biolegend |
| PerCP-Cy5.5 | CD56 | HCD56 | Biolegend |
| BV605 | CD14 | M5E2 | Biolegend |
| APC | CD16 | 3G8 | Biolegend |
| APC-H7 | HLA-DR | G46-6 | BD Pharmingen |
| BV711 | CCR7 | G043H7 | Biolegend |
| BV421 | CD1c | L161 | Biolegend |
| PE | CD163 | M80 | Biolegend |
