# Supplementary figures and images for: Novel translational model of resolving inflammation triggered by UV‐killed E. coli
Source: J Pathol Clin Res. 2016 May 4;2(3):154–65. doi: 10.1002/cjp2.43 (PMC4958736; doi:10.1002/cjp2.43)

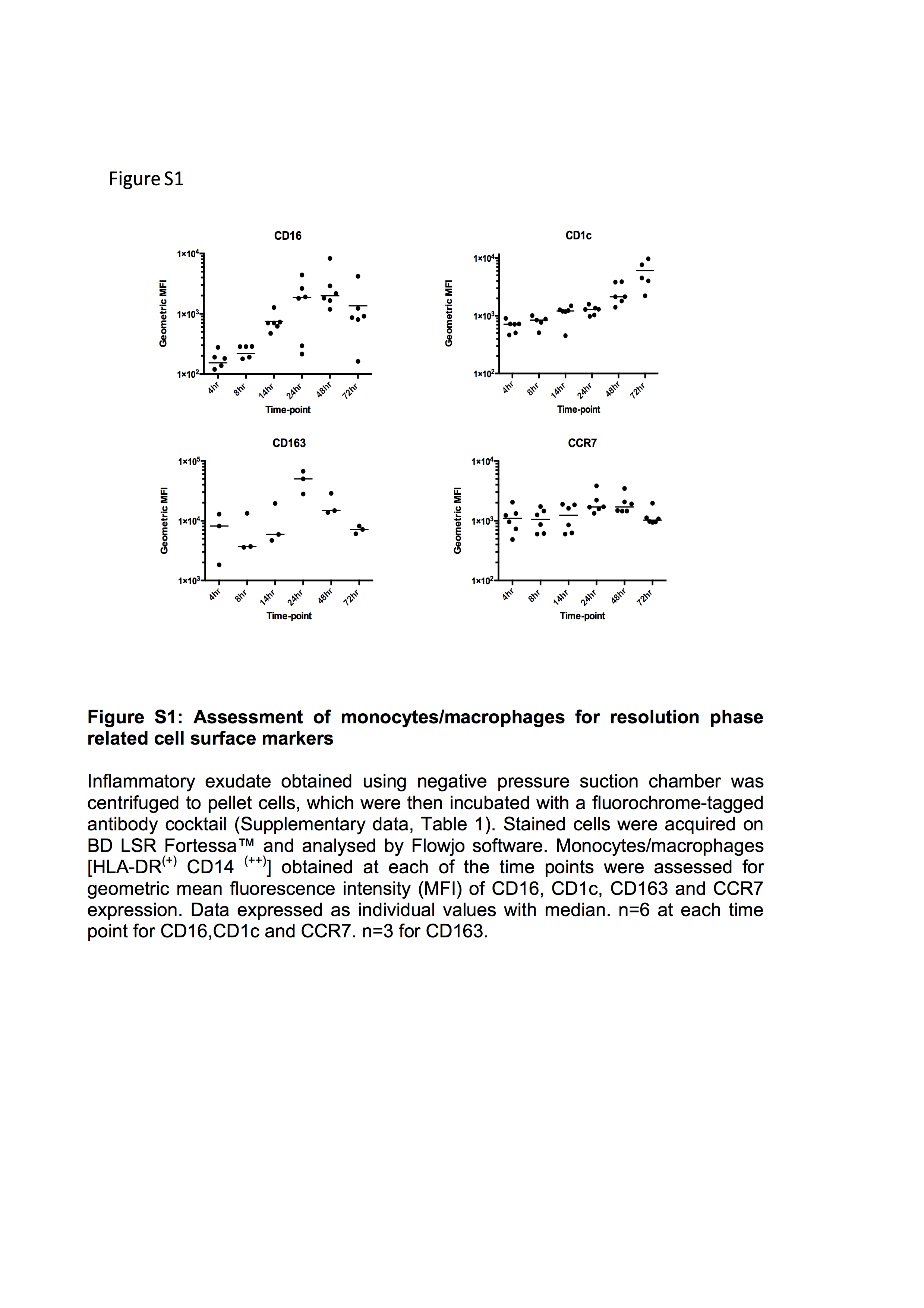

Supplement: Supplementary file 2 — Figure S1. Assessment of monocytes/macrophages for resolution phase related cell surface markers [file CJP2-2-154-s001.tiff]

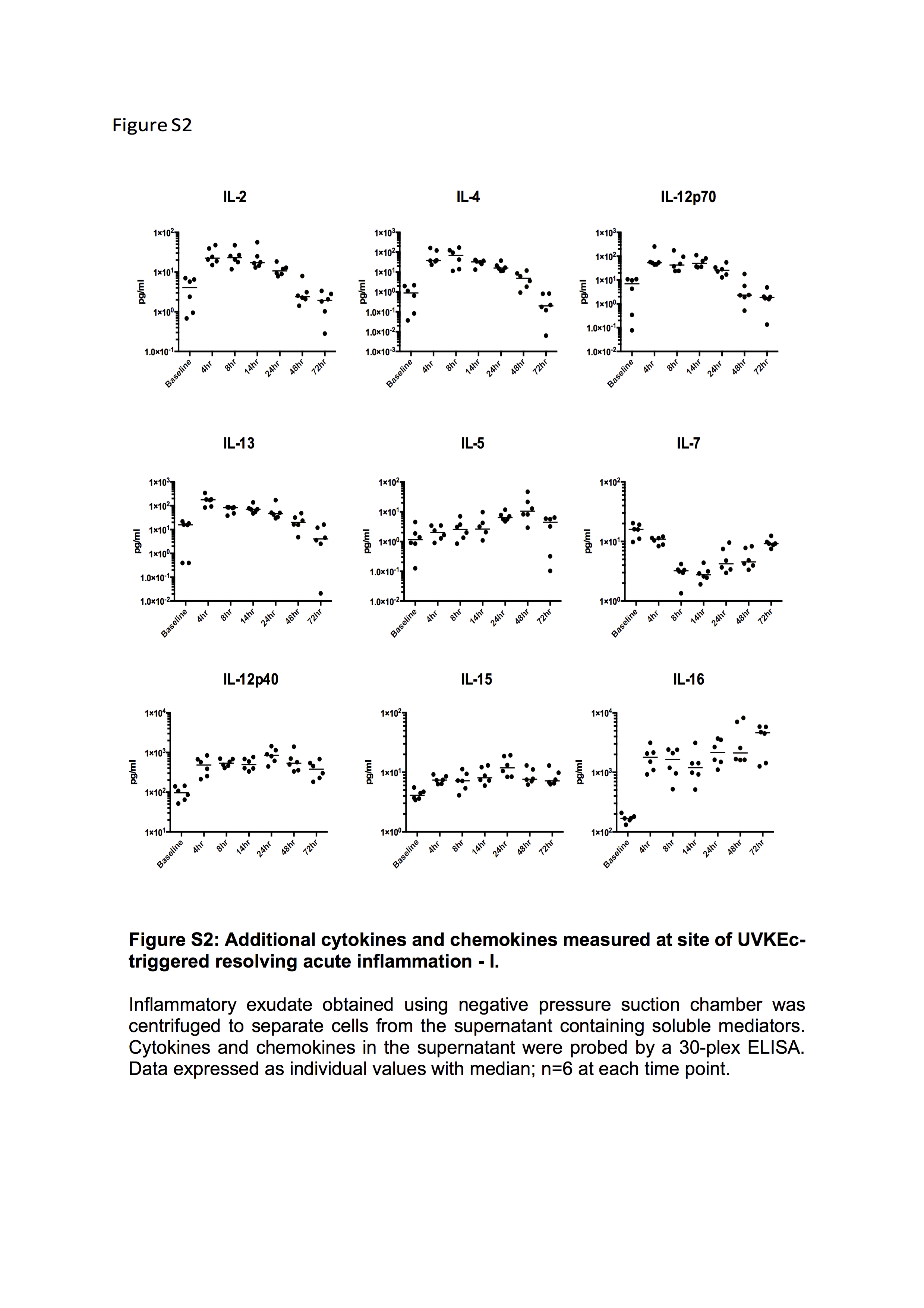

Supplement: Supplementary file 3 — Figure S2. Additional cytokines and chemokines measured at the site of UVKEc‐triggered resolving acute inflammation ‐ I [file CJP2-2-154-s002.tiff]

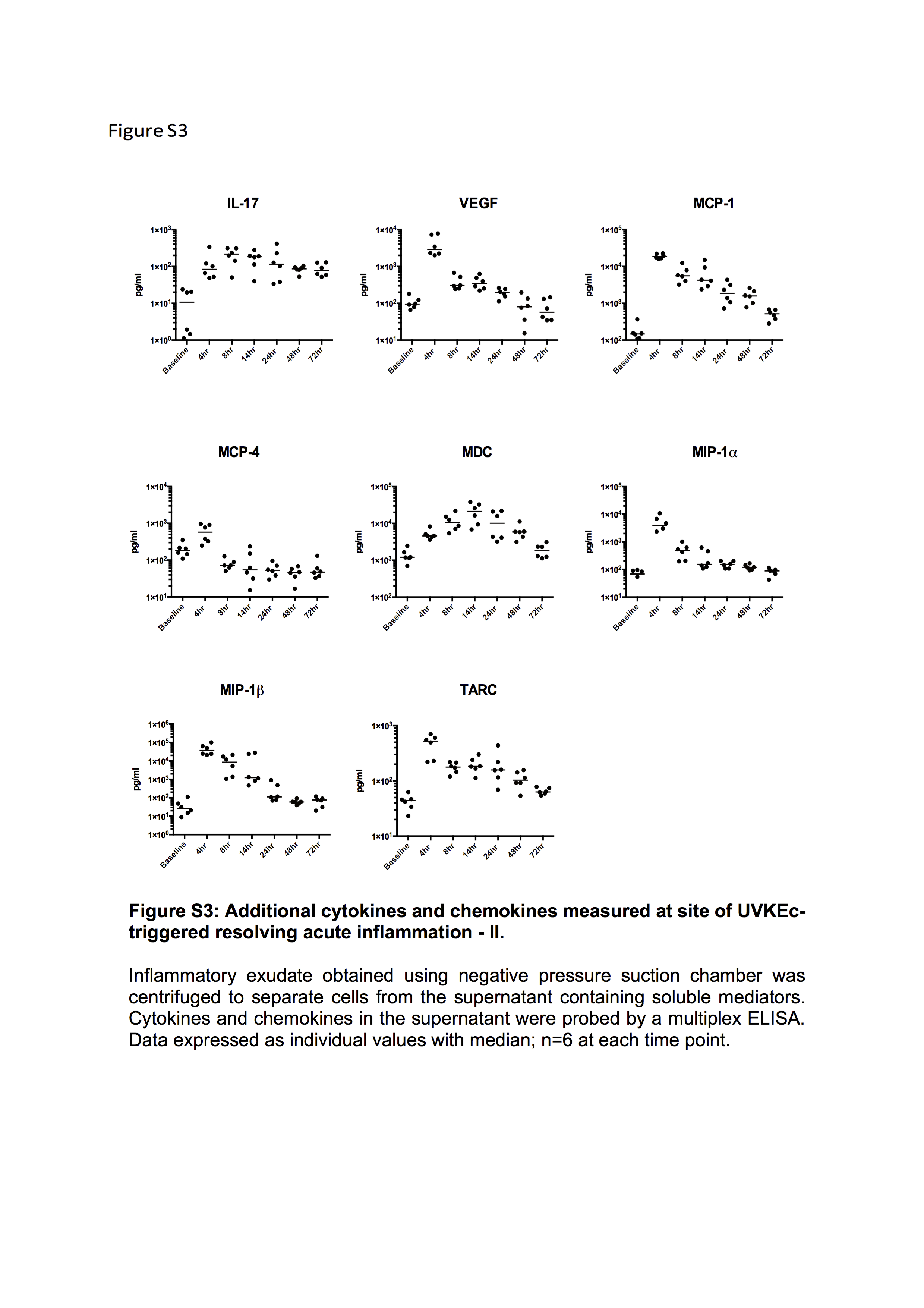

Supplement: Supplementary file 4 — Figure S3. Additional cytokines and chemokines measured at the site of UVKEc‐triggered resolving acute inflammation ‐ II [file CJP2-2-154-s003.tiff]

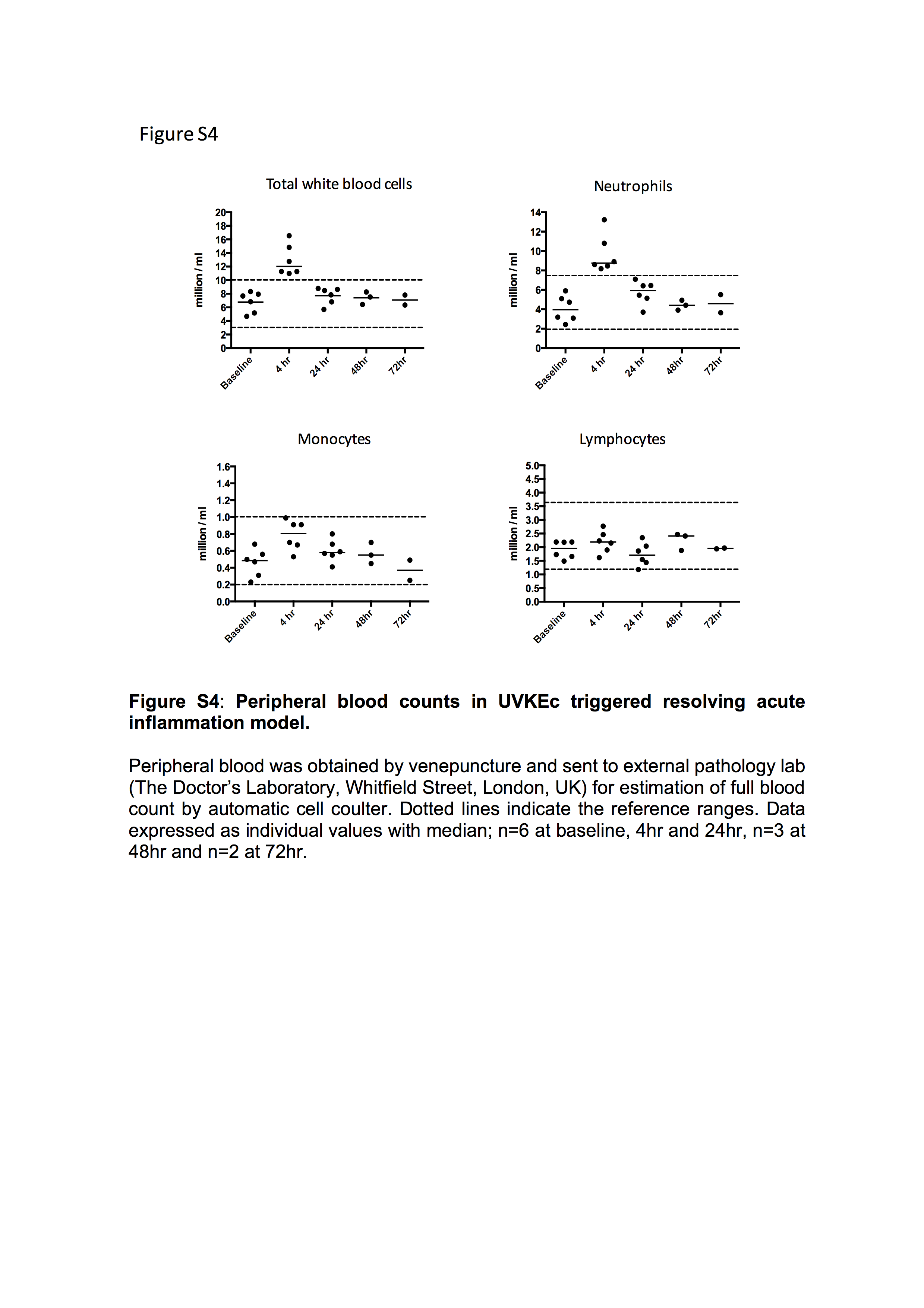

Supplement: Supplementary file 5 — Figure S4. Peripheral blood counts in UVKEc‐triggered resolving acute inflammation model [file CJP2-2-154-s004.tiff]

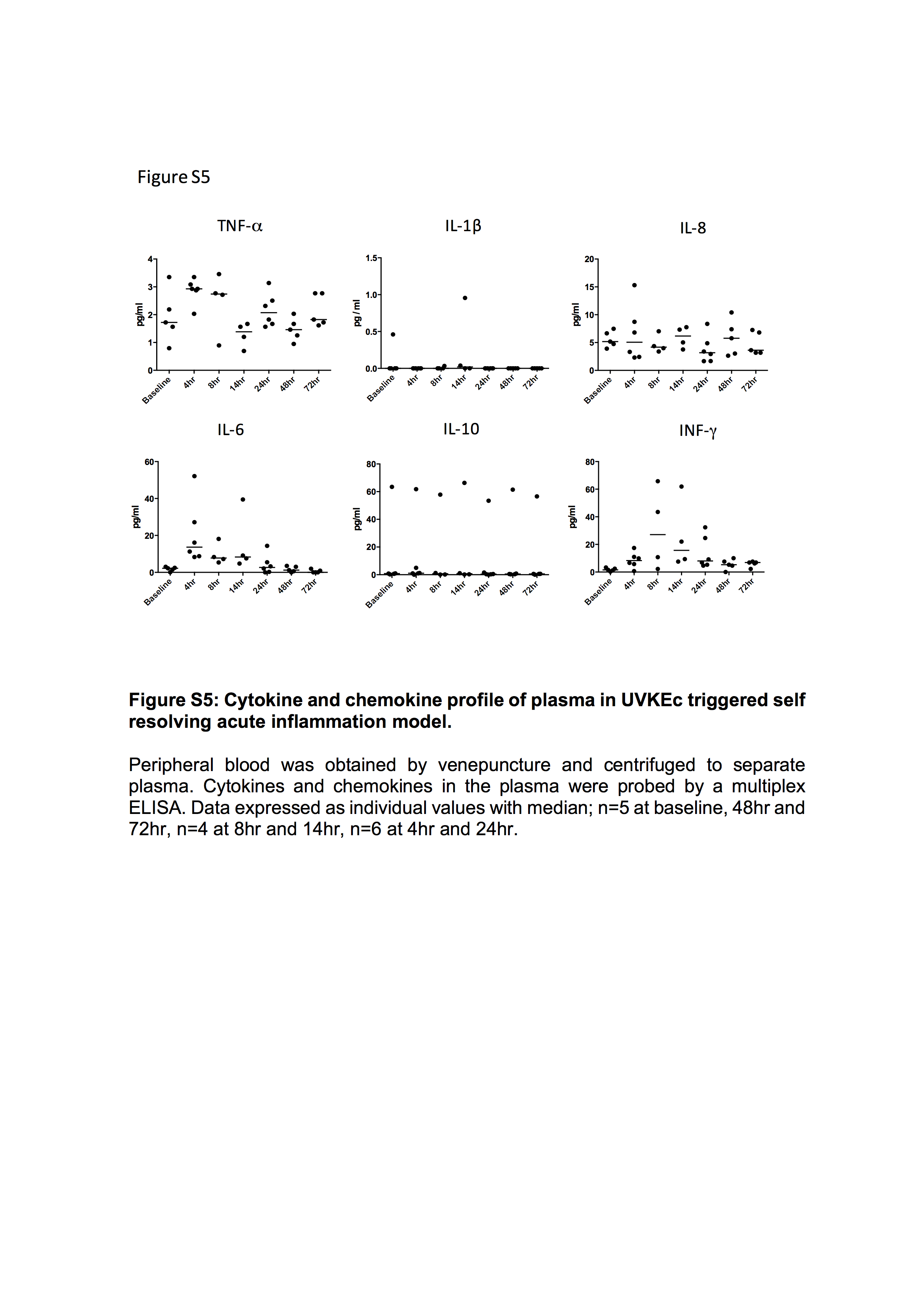

Supplement: Supplementary file 6 — Figure S5. Cytokine and chemokine profile of plasma in UVKEc‐triggered self‐resolving acute inflammation model [file CJP2-2-154-s005.tiff]
